# Non-criteria antiphospholipid antibodies and pediatric rheumatic disease: a case series

**DOI:** 10.1186/s12969-022-00732-4

**Published:** 2022-08-20

**Authors:** Shawn A. Mahmud, Danielle R. Bullock, Colleen K. Correll, Patricia M. Hobday, Mona M. Riskalla, Richard K. Vehe, Bryce A. Binstadt

**Affiliations:** 1 Department of Pediatrics, Division of Pediatric Rheumatology, Allergy & Immunology, University of Minnesota, AO-10 Academic Office Building, 2414 S. 7th Street, Minneapolis, MN USA; 2 Center for Immunology, University of Minnesota, Minneapolis, MN USA

**Keywords:** Non-criteria antiphospholipid antibodies, Antiphospholipid syndrome, Rheumatic disease, Lupus anticoagulant

## Abstract

**Background:**

Non-criteria antiphospholipid antibodies (NC-aPL) are a relatively undefined subgroup of antiphospholipid antibodies (aPL). Knowledge about NC-aPL in adults is limited and even less is known in pediatric patients. Routine tests for antiphospholipid syndrome (APS)—a clinical state marked by the presence of aPL in association with vascular thrombosis—usually include lupus anticoagulant (LAC), anti-cardiolipin (aCL) and -beta-2 glycoprotein I (aβ2GPI). LAC is a functional screen for prothrombotic aPL, while the latter tests identify specific autoantibodies. Specific targets of NC-aPL include, but are not limited to, phosphatidylethanolamine, phosphatidylserine, and prothrombin.

**Presentation of cases:**

We present single-center data from eight pediatric patients with NC-aPL identified during a three-year period. All patients had presenting features raising suspicion for APS. Most patients were female with a primary rheumatic disease. One patient had a stroke. Another patient had alveolar hemorrhage and pulmonary hypertension. Raynaud’s phenomenon, rashes involving distal extremities, and headaches were common. Most patients had a positive LAC, yet their routine aPL tests were negative, prompting testing for NC-aPL.

**Conclusions:**

Our findings suggest NC-aPL are associated with typical signs and symptoms of APS in pediatric patients. Pediatricians and pediatric subspecialists should consider NC-aPL when clinical suspicion is high and routine aPL tests are negative, particularly when LAC is positive. While guidelines for NC-aPL do not yet exist for children or adults, these autoantibodies have pathogenic potential. Actionable items could include evaluation for the presence of other (primary) rheumatic diseases, and consultation with hematologists and/or obstetricians regarding anticoagulation/platelet inhibition and thrombosis education. Future guidelines regarding NC-aPL will only be generated by gathering more data, ideally prospectively.

## Background

Antiphospholipid antibodies (aPL) are autoantibodies that bind to phospholipid-binding proteins and can provoke tissue pathology [1–5]. The diagnosis of antiphospholipid syndrome (APS) is reserved for patients who develop vascular thrombosis in association with aPL or a lupus anticoagulant (LAC), which screens indirectly for the presence of aPL. APS can be a primary condition or can occur secondary to rheumatic diseases such as systemic lupus erythematosus (SLE). Both primary and secondary APS can also feature non-thrombotic manifestations, including Raynaud’s phenomenon, livedoid and/or vasculitic rashes, headaches and other neurologic manifestations, and cardiac valve inflammation [6–13].

The most common aPL tested in clinical laboratories are IgM and IgG anti-cardiolipin (aCL) and anti-beta 2 glycoprotein-I (aβ2GPI). Medium- or high-titers of these autoantibodies—when detected two or more times at least 12 weeks apart—can fulfill the laboratory criteria of the most highly utilized classification criteria for APS in adult patients [14]. Development of revised criteria are underway [15]. The presence of aPL can also be inferred indirectly by LAC testing. A positive LAC detects in vitro inhibition of the phospholipid-dependent steps of the coagulation cascade. This nomenclature is often confusing to clinicians since these autoantibodies are pro-coagulant in vivo but prolong this coagulation test in vitro. It is possible to have a positive LAC with negative aCL and aβ2GPI. In these cases, non-criteria aPL (NC-aPL) may be responsible for LAC positivity. NC-aPL bind to other unique phospholipid-associated targets such as phosphatidylethanolamine (PE), phosphatidylserine (PS), prothrombin (PT), and others [16–21]. NC-aPL are not well characterized even in adult patients. These antibodies are referred to as ‘non-criteria’ because there are insufficient prospective data available to justify including them in common classification criteria for APS [16]. Even less is known about NC-aPL in the pediatric population.

In the following single-center case series, NC-aPL testing was obtained in individual patients due to clinical suspicion for APS, particularly when aCL and aβ2GPI antibodies were negative in the setting of a positive LAC. Here we review the clinical presentation, laboratory and other diagnostic evaluation, and management of eight pediatric patients with NC-aPL.

## Methods

All included patients were convenience samples. However, we also performed a database search of the electronic medical record at the University of Minnesota to ensure that all pediatric patients with positive NC-aPL tests were captured within the preceding 36 months. The University of Minnesota Institutional Review Board deemed this study exempt from full review. NC-aPL testing was not available at our institution, so samples were sent to ARUP laboratories (“Non-Criteria Antiphospholipid Syndrome Antibodies Panel” Ref# 2012729) and Mayo Clinic Laboratories (“Anti-Phosphatidylethanolamine Panel” Ref# FPHET). Tests for LAC, aCL, and aβ2GPI were performed in-house using commercially available, Clinical Laboratory Improvement Amendments (CLIA)-certified ELISA kits read on a BioRad BioPlex plate reader (Herculus, CA) in the University of Minnesota Specialty Protein Core Laboratory. LAC testing in the University of Minnesota Specialty Coagulation Laboratory is based on dilute Russel’s Viper Venom test (dRVVT). INR, PTT, and dRVVT tests are conducted and results are reported as a dRVVT normalized ratio. PTT testing and dRVVT reagents were manufactured by Stago (Parsippany, NJ). Normal human plasma was obtained from Precision Biologics (Dallas, TX). Internal QC ranges were re-established with each lot of normal human plasma. Samples with a dRVVT normalized ratio of ≥ 1.21 were considered positive. There are no pediatric-specific controls or age cut-offs in this assay.

## Presentation of Cases

**Fig. 1:**
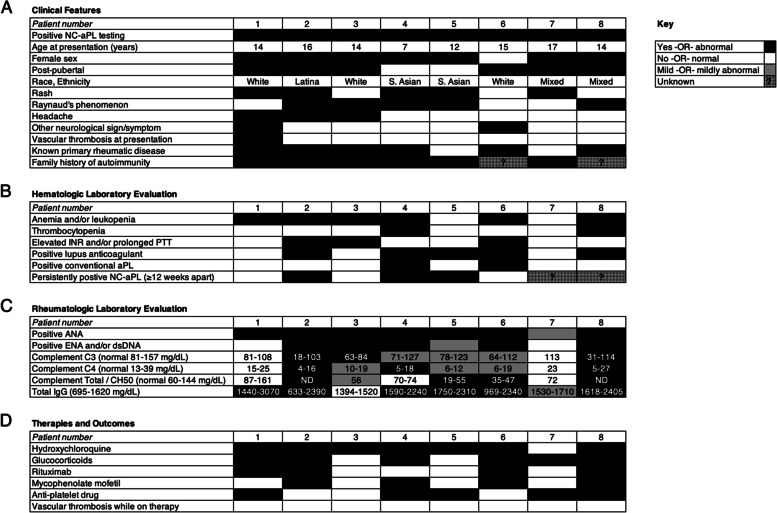
Clinical characteristics (**A**), hematologic (**B**) and rheumatologic (**C**) laboratory evaluation, and therapies and outcomes (**D**) observed in patients with NC-aPL. Interpretation key is shown to the right. Ranges of values for complement C3, C4, CH50, and total IgG for each individual patient are shown in cells in (**C**). Abbreviations: INR: international normalized ratio, PTT: partial thromboplastin time, ANA: antinuclear antibodies, ENA: antibodies to extractable nuclear antigens, dsDNA: anti-double stranded DNA

Key clinical features of the patients are summarized in Fig. 1A. Seven of the eight patients (88%) were female. The age at time of presentation ranged from 7–17 years (avg. 13.6) and most of the patients (75%) were post-pubertal at the time of presentation. Patients were of diverse racial and ethnic backgrounds and most had a family history of autoimmunity in either a first- or second-degree relative (Fig. 1A). Notable features at the time of NC-aPL testing included dermatologic, vascular, hematologic, and neurologic findings. Rashes were present in half of the patients, including livedoid rash in two patients and non-blanching purpura of the extremities in two others. Most patients had Raynaud’s phenomenon—a common APS-associated manifestation caused by vasospasm of digital arteries [22–24]. Neurologic findings included chronic headaches (*n* = 3), absence seizures (*n* = 1), and chorea and cerebellar stroke (*n* = 1; Fig. 1A). Only one patient had a documented thrombotic event (the cerebellar stroke). This patient also had mitral valve thickening and regurgitation, but the stroke was not suspected to be embolic based on MRI appearance. Evaluation for inherited thrombophilia in the patient who had a stroke was negative. One patient with concomitant systemic lupus erythematosus (SLE) developed alveolar hemorrhage and pulmonary hypertension believed to be secondary to pulmonary capillaritis; however, it was unclear if these were manifestations of SLE, NC-aPL, or both.

Laboratory and other diagnostic evaluations (Fig. 1B and C) showed that six of eight patients had anemia, leukopenia, and/or thrombocytopenia. Most patients had abnormal coagulation function (elevated and/or prolonged INR and/or PTT, respectively). Strikingly, while most patients had a positive LAC, 75% had negative aCL and aβ2GPI antibodies. Although the same NC-aPL panel was not sent on all patients, most had seropositivity to anti-PS and/or anti-PS/PT.

Pediatric-specific APS criteria have not yet been clearly established; however, none of the patients in this series met adult classification criteria for APS. Only one patient had a history of thrombosis, which is a requirement in the classification criteria for APS [14]. This patient was treated with the B cell-depleting monoclonal antibody, rituximab, prior to repeating NC-aPL testing; upon re-testing more than 12 weeks later, the originally detected NC-aPL were no longer present. We therefore suspect that treatment with rituximab likely ablated NC-aPL positivity. Even if NC-aPL were currently included in consensus laboratory classification criteria for APS, this patient would not have fulfilled laboratory criteria because the antibodies were not durably detectable (that is, present on at least two occasions, ≥ 12 weeks apart). Infections can commonly cause a transient positivity to aPL. No infectious causes were identified in the case of transient positivity to NC-aPL in this series.

At the time NC-aPL were tested, a clear rheumatic disease was present in five of eight patients. After additional follow-up visits, one additional patient had evolved to demonstrate a clear primary rheumatic disease. The primary rheumatic diseases in these patients included SLE (*n* = 4), mixed connective tissue disease (*n* = 1), and Sjogren syndrome (*n* = 1) (Fig. 1A). Therefore, these six patients were suspected to have NC-aPL secondary to a known rheumatic disease. The other two patients had no discernable primary rheumatic disease associated with the presence of NC-aPL. Most patients had hypergammaglobulinemia and many had hypocomplementemia (Fig. 1C), which was not surprising given the primary rheumatic diseases present in this series of patients. In general, complements C3 and C4 began to increase and total IgG decreased after starting therapies in patients in which these levels were abnormal at the time of presentation and NC-aPL detection (data not shown).

Therapy was primarily directed at treating the underlying rheumatic diseases present in these patients, and a high degree of commonality in the treatments utilized was observed (Fig. 1D). Almost all patients were treated with hydroxychloroquine, a commonly prescribed antimalarial drug with immunomodulatory and antiplatelet effects [25–30]. Several patients also received glucocorticoids, mycophenolate, and rituximab. Five of eight patients were treated with conventional antiplatelet drugs, such as aspirin or clopidogrel. No patients were treated with long-term heparin or warfarin, although the former was utilized in select patients when hospitalized with other obvious risk factors for thrombosis (e.g., hemoconcentration, nephrotic range proteinuria, immobilization). Decisions regarding initiation of antiplatelet drugs and/or short-term anticoagulation were made in conjunction with pediatric hematologists. None of the patients in this series has suffered thrombotic or bleeding events after the initiation of therapy; however, the maximum period of observation of any individual patient is 36 months.

## Discussion and conclusions

These data suggest that the clinical features associated with NC-aPL positivity in pediatric patients are similar to those seen in patients with positive conventional aPL tests or APS, including livedoid and/or vasculitic rashes, Raynaud’s phenomenon, CNS symptoms (headaches, seizures, strokes, and chorea), cardiac valve inflammation, and cytopenias [6–12]. While non-thrombotic manifestations that are commonly associated with aPL positivity and APS were seen in this series, thrombosis was notably rare.

NC-aPL positivity in these patients was most commonly observed in association with a well-defined primary rheumatic disease (i.e., NC-aPL positivity was most often presumed to be secondary to a known rheumatic disease). Amongst these diseases, SLE stood out as the most common. As such, many patients exhibited hypergammaglobulinemia, positive anti-nuclear antibodies (ANA), positive anti-double stranded DNA (dsDNA) and/or positivity to extractable nuclear antigens (ENA). However, it is important to point out that pediatric rheumatologists caring for patients either in outpatient clinics or during inpatient consultation identified all the patients in this series. Therefore, this series likely exhibits selection bias for patients with a primary rheumatic disease.

We hypothesize that in comparison to adult patients with NC-aPL or conventional aPL, thrombosis is less likely to be a frequent manifestation in pediatric patients [31]. Studies of developmental hemostasis have demonstrated that the levels of many procoagulant factors (FII, FV, FVII) increase throughout childhood and more so into adolescence after puberty [32–34]. Thrombosis risk is an age-dependent phenomenon [35–38]. We hypothesize that risk of thrombosis secondary to NC-aPL is also likely age-dependent. However, many pediatric/adolescent patients may not yet have had sufficient time to accumulate additional risk factors such as hyper-estrogenic states (e.g., pregnancy, obesity), atherosclerotic cardiovascular disease, peripheral arterial disease, kidney disease, and others. It is therefore important that pediatric rheumatologists recognize the non-thrombotic manifestations of NC-aPL and aPL in addition to assessing a patient’s broader risks for thrombosis.

Our case series further suggests that NC-aPL may be pathogenic and associated with signs and symptoms of APS. Prospective data regarding these autoantibodies and the risk of thrombosis, association with non-thrombotic manifestations of APS, and response to therapy are needed. Pediatric rheumatologists should consider ordering NC-aPL testing when there is strong clinical suspicion for APS and when routine tests for aCL and aβ2GPI are negative, particularly when LAC is positive. A positive LAC is not highly specific. It may suggest the presence of aPL or NC-aPL. However, it may be positive in genetic or acquired deficiencies of factors in the coagulation cascade (FI, FII, FV, FVIII or FX). Similarly, patients with highly elevated levels of FVIII, as can be seen in active SLE or acute infection may have a false negative LAC [39]. Identification of positive NC-aPL should lead to further evaluation to determine if an underlying primary rheumatic disease is also present. Moreover, hematology consultation may be warranted in patients with NC-aPL to consider antiplatelet drugs and/or anticoagulation. Patients with NC-aPL should be educated about thrombosis and its risk factors, and about potential implications associated with the presence of these autoantibodies later in life, especially during pregnancy or if starting estrogen-containing contraceptive agents.

Evidence-based recommendations for the diagnosis and management of pediatric APS with patients with conventional aPL are evolving [40]. However, guidelines for diagnosing APS in pediatric patients based on NC-aPL positivity do not currently exist, nor are there guidelines for how to manage patients with NC-aPL without a history of thrombosis. However, through future prospective studies, guidelines may be developed that are applicable to pediatric patients.

## Data Availability

The datasets analyzed during the current study are available from the corresponding author upon reasonable request.

## Abbreviations

NC-aPL: Non-criteria antiphospholipid antibodies
aPL: Antiphospholipid antibodies
APS: Antiphospholipid syndrome
LAC: Lupus anticoagulant
aCL: Anti-cardiolipin
aβGPI: Anti-β2 glycoprotein I
SLE: Systemic lupus erythematosus
PE: Phosphatidylethanolamine
PS: Phosphatidylserine
PT: Prothrombin
ANA: Anti-nuclear antibodies
dsDNA: Double stranded DNA
ENA: Extractable nuclear antigens
